# Characterization of neopeptides in equine articular cartilage degradation

**DOI:** 10.1002/jor.22963

**Published:** 2015-07-07

**Authors:** Mandy Jayne Peffers, David James Thornton, Peter David Clegg

**Affiliations:** ^1^ Department of Musculoskeletal BiologyInstitute of Ageing and Chronic DiseaseUniversity of Liverpool, LeahurstChester High RoadNestonWirralCH64 7TEUK; ^2^ Wellcome Trust Centre for Cell Matrix ResearchFaculty of Life SciencesMichael Smith BuildingOxford RoadManchesterM13 9PTUK

**Keywords:** osteoarthritis, neopeptides, cartilage, equine

## Abstract

Osteoarthritis is characterized by a loss of extracellular matrix that leads to cartilage degradation and joint space narrowing. Specific proteases, including the aggrecanases ADAMTS‐4 and matrix metalloproteinase 3, are important in initiating and promoting cartilage degradation in osteoarthritis. This study investigated protease‐specific and disease‐specific cleavage patterns of particular extracellular matrix proteins by comparing new peptide fragments, neopeptides, in specific exogenous protease‐driven digestion of a crude cartilage proteoglycan extract and an in‐vitro model of early osteoarthritis. Additionally, equine cartilage explants were treated with interleukin‐1 and the media collected. Proteolytic cleavage products following trypsin digestion were then identified using tandem mass spectrometry. Complete sequences of proteolytically cleaved neopeptides were determined for the major cartilage proteoglycans aggrecan, biglycan, decorin, fibromodulin plus cartilage oligomeric matrix protein. The generation of neopeptides varied with enzyme specificity; however, some peptides were common to all samples. Previous known and novel cleavage sites were identifies. The identification of novel peptide fragments provides a platform for the development of antibodies that could assist in the identification of biomarkers for osteoarthritis (OA), as well as the identification of basic biochemical processes underlying OA. © 2015 The Authors. *Journal of Orthopaedic Research* Published by Wiley Periodicals, Inc. on behalf of Orthopaedic Research Society. J Orthop Res 34:106–120, 2016.

The unique load‐bearing properties of articular cartilage are dependent upon its structural composition and organization, particularly the interactions between collagens and proteoglycans of the extracellular matrix (ECM).^1^ In normal physiology, these matrix macromolecules are turned over by chondrocytes embedded within the cartilage.

Progressive degeneration of articular cartilage, including proteolysis‐driven degradation, leads to joint pain and dysfunction that is clinically identified as osteoarthritis (OA). Under normal circumstances, there is an equilibrium between matrix deposition and degradation; however, this equilibrium is disrupted in OA, leading to the excessive degradation of matrix and progressive loss of important matrix components, such as collagens and proteoglycans.^2^, ^3^ Furthermore, intact and fragmented ECM peptides, produced following degradation, affect chondrocyte function through integrin receptor signaling.^4^

Studies have identified peptide fragments (neopeptides) of the ECM constituents in cartilage metalloprotease digests,^5^ tendon ageing^6^ and disease,^6^, ^7^ and OA.^8^ In cytokine‐stimulated models of OA, the breakdown of cartilage is stimulated by a series of proteolytic enzymes through the upregulation of metalloproteinase.^9^ Initially, aggrecan is fragmented and released from cartilage in OA, followed by other molecules, such as cartilage oligomeric matrix protein (COMP), fibromodulin, and collagens.^10^ Furthermore, these neopeptides can be used as biochemical markers.

Members of the matrix metalloproteinases (MMPs) and disintegrin and metalloproteinase with thrombospondin type I motifs (ADAMTS) families of enzymes are important in cartilage matrix degradation in OA.^11^ MMP‐3 is one of the most highly expressed proteases in cartilage capable of degrading proteoglycans, including aggrecan,^5^ as well as activating procollagenases. ADAMTS‐4 is an important enzyme in the pathogenesis of OA as demonstrated by its high aggrecanase activity in OA cartilage, and localized expression in the areas of aggrecan degradation.^12^ Both ADAMTSs and MMPs are involved in the cleavage of other ECM proteoglycans, including aggrecan,^13^ biglycan^14^, ^15^ and decorin,^16^ fibromodulin,^17^ and COMP.^18^

Diagnosis of OA at an early stage is difficult as it is based on radiographic changes and clinical symptoms that occur relatively late in disease. However, the advent of advanced proteomic techniques has enabled the identification and use of protein biomarkers to become established^19^ that may aid in not only the monitoring of OA progression but also the effects of therapeutics for OA. Neopeptides provide potential biomarker candidates.

In this study, we use liquid chromatography tandem mass spectrometry (LC‐MS/MS), de‐novo sequencing, and database searching to enable an accurate and convenient workflow to identify neopeptides (in selected proteoglycans; important to cartilage biology) released by either specific protease treatment of cartilage proteoglycan extract or following cytokine stimulation of equine articular cartilage. One way to provide novel insights into development and treatment of OA is to obtain an overall understanding of cartilage degradation. This analysis was performed to increase the knowledge of molecular events associated with cartilage degradation characteristic of OA, identify peptides that may be useful as biomarkers of cartilage disease, as well as to identify novel neopeptides that could be further validated in targeted future studies.

## EXPERIMENTAL PROCEDURES

All chemicals were supplied by Sigma–Aldrich, Dorset, UK unless otherwise stated.

### Cartilage Sampling and Procurement

Samples were collected as a by‐product of the agricultural industry. Specifically, the Animal (Scientific Procedures) Act 1986, Schedule 2, does not define collection from these sources as scientific procedures. Ethical approval was, therefore, not required for this study. Full‐thickness cartilage from the entire surface of macroscopically normal metacarpophalangeal joints of three independent horses for each study (explant; mean age 7 ± 1 years, proteoglycan isolation 8.3 ± 0.6 years) was collected from an abattoir. Macroscopic scoring was undertaken using a macroscopic grading system as described previously.^20^

### Normal Equine Cartilage IL‐1β‐Treated Explant Studies

Cartilage explant studies were undertaken as previously described.^21^ The media was exchanged 48 h after initiation of treatment, and cultures harvested after 96 h. The 48‐ and 96‐h supernatant samples were pooled, thus representing the total secretome over 96 h.

### Proteoglycan‐Enriched Fraction Isolation From Cartilage

Proteoglycan was extracted from cartilage pooled from three donors using 4 M guanidinium chloride as previously described.^6^ The soluble fraction was removed following centrifugation for 15 min at 13,000*g* at 4 °C and dialyzed in a 14,000‐kD cut‐off membrane (Spectrapor, Breda, the Netherlands) for 24 h at 4 °C against 0.1 M sodium acetate, pH 6.0 in the presence of protease inhibitors. The extract was centrifuged for 15 min at 13000*g* at 4 °C. The supernatant was fractionated in an associative cesium chloride (CsCl) density gradient (starting density 1.5 g/ml) for 60 h at 100,000*g* in an ultracentrifuge (Beckman 50Ti, Gallway, Ireland). The tube was fractionated into quarters, A1–A4. The combined A1–A2 fractions, identified in previous studies as being enriched for aggrecan and small leucine‐rich proteoglycans,^22^ were retained for protease digestion and dialyzed against 0.1 M sodium acetate for 48 h at 4 °C and then against ultrapure water for 36 h at 4 °C. The samples were then lyophilized. An aliquot of each fraction was assessed for protein content using optical density of 280 nm with a Nanodrop ND‐100 spectrophotometer (Labtech, East Sussex, UK). To validate, the A1–A2 fractions were enriched for proteoglycans, glycosaminoglycan (GAG) analysis of the A1–A4 fractions was undertaken using a 1,9‐dimethyl‐methylene blue (DMMB) dye‐binding microwell spectrophotometric assay.^23^

### Protease Digestion of the Proteoglycan Extract

Aliquots of the A1–A2 extract were digested in protease digestion buffer (50 mM Tris HCl, 100 mM NaCl, 10 mM CaCl_2_, pH 7.5) with either 0.05 nmol human recombinant MMP‐3 catalytic domain (Calbiochem, La Jolla, CA) for 20 h at 37 °C or with 0.014 nmol truncated human recombinant ADAMTS‐4 (Calbiochem) for 7 h at 37 °C. Times were chosen based on preliminary studies (data not shown). A control for each protease was incubated under the same conditions in the presence of the recombinant protein formulation buffer. The enzymatic digestion reactions were stopped by addition of EDTA.

### Deglycosylation of the Proteoglycan Extract and Immunoblot Analysis

A1–A2 extracts crude proteoglycan extracts (CPE) before and after protease digestion were deglycosylated as previous described.^13^ Deglycosylated samples of the CPE, equivalent to 5 µg GAG were applied to the nitrocellulose membrane. Samples were prepared for immunoblotting as described.^24^ The membrane was probed overnight at 4 °C with the following antibodies in TBS‐T containing 5% milk: mouse monoclonal to aggrecan ARGxx (BC‐3) (Abcam, Cambridge, UK) (1:100 dilution), mouse monoclonal to aggrecan DIPEN (MD Bioproducts, Minneapolis, MN) (1:100 dilution), and rabbit polyclonal to aggrecan (Abcam) (1:1000). The following secondary peroxidise conjugated antibodies were used: goat anti‐mouse IgG and goat anti‐rabbit IgG both at 1:1000 dilution (Abcam).

### In‐Solution Tryptic Digestion

Samples of cartilage supernatant from the explant experiments and proteoglycan extract from the protease digestion experiments were trypsin digested as described previously.^6^ CPE samples were desalted and purified using C_18_ resin in the form of a ZipTip® (Merck Millipore, Billerica, MA).

### LC‐MS/MS Analysis

LC‐MS/MS analysis was performed using NanoAcquity™ Ultraperformance LC (Waters, Manchester, UK) on line to an LTQ‐Orbitrap Velos (Thermo‐Fisher Scientific, Hemel Hempstead, UK). Aliquots of tryptic peptides equivalent to 250 ng were loaded as previously described.^21^

### Neopeptide Identification

For neopeptide identification, raw spectra were converted to mascot generated files (mgf) using Proteome Discoverer software (Thermo, Hemel Hempstead, UK). The resulting mgf files were searched against the Unihorse database using an in‐house Mascot^25^ server (Matrix Science, London, UK). Search parameters used were as follows: enzyme; none, peptide mass tolerances 10 ppm, fragment mass tolerance of 0.6Da, 1+, 2+, and 3+ ions, missed cleavages; 1, and instrument type ESI‐TRAP. Modifications included were as follows: fixed; carbamidomethyl cysteine and variable; oxidation of methionine. The probability that a match was correct (*p *< 0.05) was determined using the Mascot‐derived ion score, where *p* was the probability that the observed match was a random event. For reasons of economy and to have confidence in our analysis, we only included neopeptides in the results if they were present in treated samples exclusively as identified by Mascot. Patterns of fragmentation were determined for aggrecan, biglycan, decorin, fibromodulin, and COMP.

In addition, raw data files were loaded into PEAKS® Studio 6.0 (Bioinformatics Solutions, Inc., Waterloo, Canada) and de‐novo sequencing and protein identification performed. Estimate FDR function was used in order to create a “decoy fusion” database based on the Ensembl database for horse (*Equus caballus*; EquCab2.56.pep, (ftp://ftp.ensembl.org/pub/current_fasta/equus_caballus/pep/). Results generated using PEAKS® Studio was manually curated against the Mascot search engine results. A 10lgP score of >20 was considered as significant. Mass spectrometry data is available in PRIDE are available in the PRIDE database (http://www.ebi.ac.uk/pride/) at the European Bioinformatics Institute under accession numbers 19447–19479.

## RESULTS

### Production of a Proteoglycan‐Enriched Fraction by Cesium Chloride Density Gradient‐Ultracentrifugation

**Figure 1:**
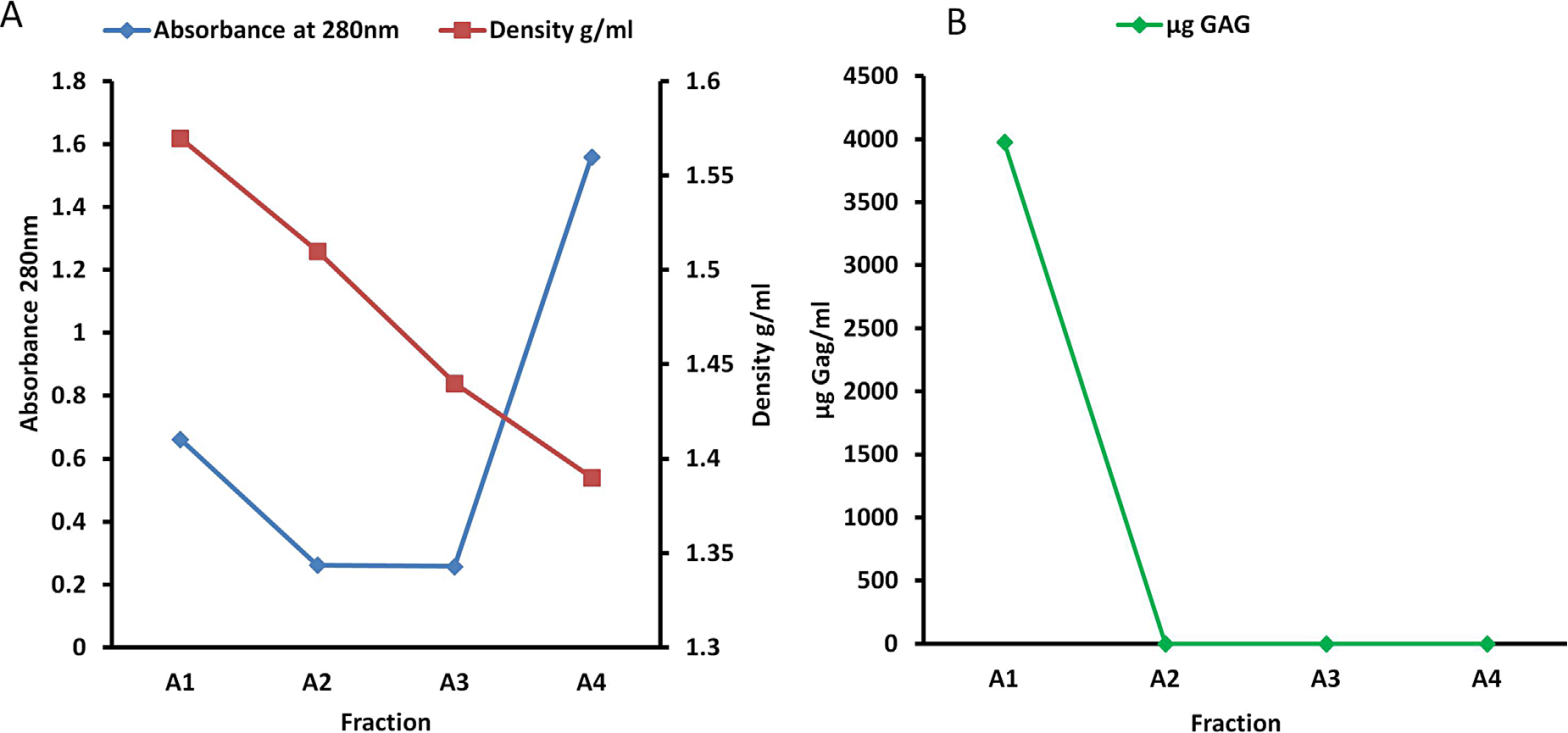
Guanidinium chloride extraction and CsCl centrifugation produced a proteoglycan‐enriched fraction of three pooled samples of equine cartilage. Solid CsCl was added to extracted soluble cartilage proteins at a starting density of 1.5 g/ml. After centrifugation, the tubes were fractionated into four equal fractions and the density of each fraction was measured. Fractions were then measured for (A) protein absorbance at 280 nm, and assayed to determine (B) GAG concentrations of each fraction. Fractions A1 and A2 were then pooled for further work.

In this study, we were interested in identifying potential cleavage sites in cartilage proteoglycans and COMP. Therefore. the CPE, which was also found to be abundant in COMP, was purified using cesium chloride density gradient centrifugation. Proteoglycans were found, as validated by the GAG assay results at the expected density of 1.3–1.5 g/ml (Fig. 1). Proteins were predominantly in the A4 fraction as determined by protein absorbance (data not shown). The proteoglycan‐rich A1–A2 fractions were then pooled for further work.

### Human Recombinant Proteases Were Validated for the Use in Equine Cartilage Proteoglycan‐Enriched Fraction Digestion

**Figure 2:**
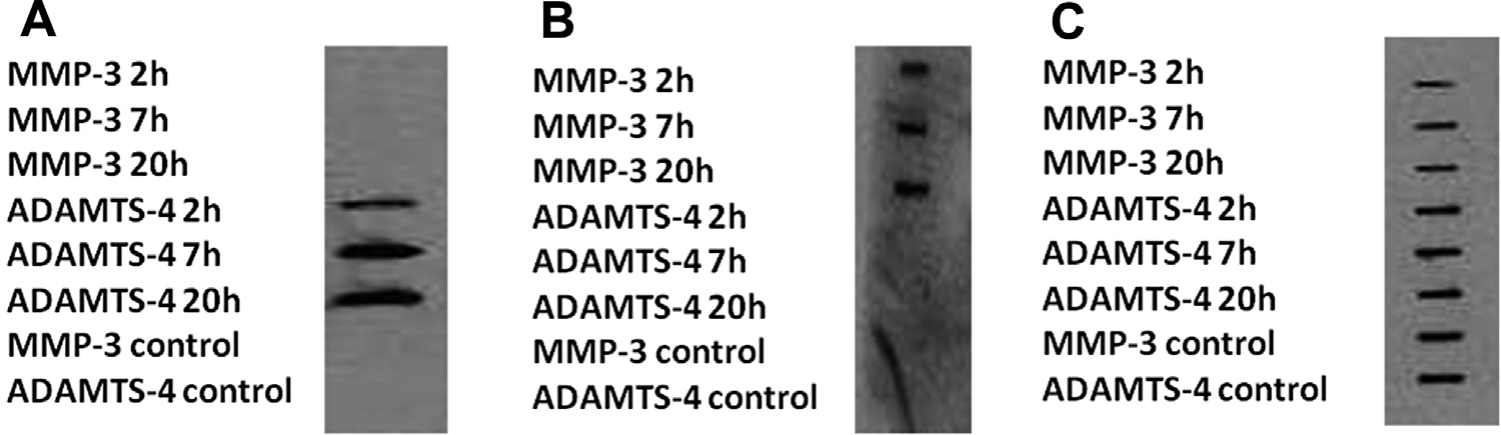
A slot blot of MMP‐3 and ADAMTS‐4 digests of cartilage proteoglycan‐enriched extracts using anti‐neoepitope antibodies reveals that human recombinant proteins are active against equine aggrecan. Proteoglycan‐enriched fractions were extracted from pooled equine cartilage of three donors and analyzed both before and after ADAMTS‐4 or MMP‐3 digestion using the following antibodies: (A) anti‐ARGxx, (B) anti‐DIPEN, and (C) Anti‐aggrecan. Prior to ADAMTS‐4/MMP‐3 digestion, only bands identifying intact aggrecan were seen. Following ADAMTS‐4 digestion at all incubation times, ADAMTS‐4‐derived degradation products were evident. Similar results were identified following MMP‐3 digestion.

As equine recombinant proteases relevant to this study were not freely available, human recombinant proteases were used. Crude proteoglycan extracts were analyzed before (control) and after MMP‐3 or ADAMTS‐4 digestion. Immunoblotting was undertaken with neoepitope antibodies. Neoepitopes are defined as part of a molecule that is the target of an immune response. In this case, the target is a peptide fragment. Immunoblotting using anti‐ARGxx antibody (ADAMTS‐4 derived neoepitope), anti‐DIPEN antibody (MMP‐3 derived neoepitope), and anti‐aggrecan revealed intact aggrecan, as well as degradation products consistent with the activity of human recombinant proteases on equine aggrecan. ADAMTS‐4 proteolysis (but as expected, not MMP‐3) produced a product identified by the aggrecanase derived antibody ARGxx (Fig. 2A) and MMP‐3 digestion produced a product identified by the DIPEN antibody (Fig. 2B).

### Identification of Neopeptides in Protease‐Treated Proteoglycan‐Enriched Fraction

**Figure 3:**
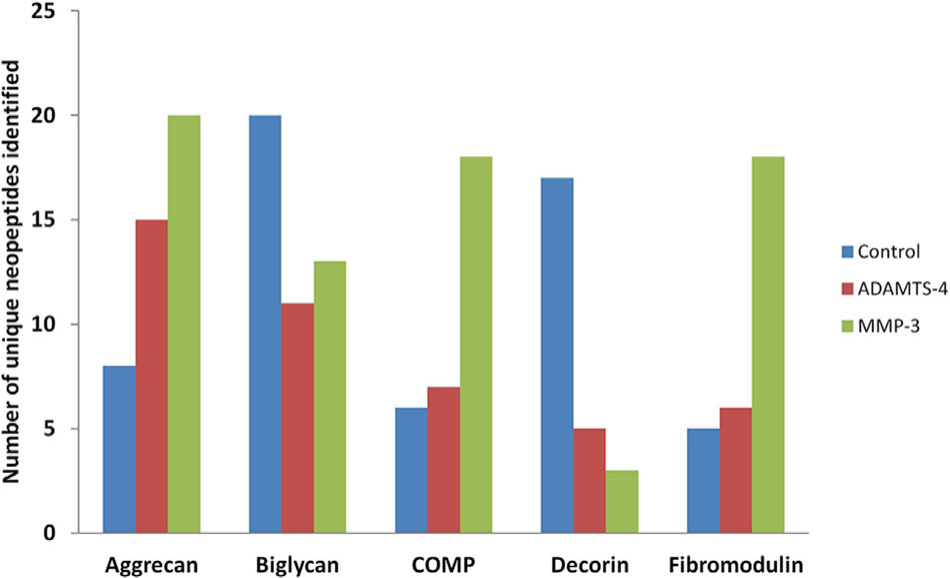
Digestion of crude equine cartilage proteoglycan with MMP‐3 or ADAMTS‐4 produced a number of neopeptides. Histogram of neopeptides identified following a Mascot search of the Unihorse database following protease digestion with either ADAMTS‐4 or MMP‐3 or in control conditions of crude equine proteoglycan extract. Results were from the pooled proteoglycan‐rich extract from three donors analyzed on the instrument in singlicate. Peptides included in the histogram were exclusively identified in the samples assigned. The control contained digestion buffer but no exogenous protease.

Mascot identified 87 and 84 significant proteins with more than one unique peptide for the ADAMTS‐4 and MMP‐3 digests, respectively. The proteoglycans (aggrecan, biglycan, decorin, and fibromodulin) investigated in this study and COMP were in the top 10 proteins identified in all samples. Supplementary Table S1 provides detailed information on the identification of peptides mapped to each protein and corresponding Mascot scores. The numbers of neopeptides that were generated are demonstrated in Figure 3. Table 1 indicates the number of unique neopeptides identified for each protease digestion. Table 2A and B (ADAMTS‐4) and Table 3A and B (MMP‐3) demonstrate sequences of neopeptides. The number of times each neopeptide was identified varied from once (76% and 74% in ADAMTS‐4 and MMP‐3 digestion, respectively) to 33 times within a single experiment for the MMP‐3 derived biglycan neopeptide ^152^NHLVEIPPNLPSS^164^.

**Table 1:** Degradation‐Derived Neopeptides Were Identified in Crude Proteoglycan Extract and Cytokine‐Driven Cartilage Degradation Using LC‐MS/MS

	Treatment								
	ADAMTS‐4			MMP‐3			IL‐1β		
Protein	Total No. Peptides	Significant Peptides *p *< 0.05^#^	Significant 10lgP^*^	Total No. Peptides	Significant Peptides *p* < 0.05^#^	Significant 10lgP^*^	Total No. Peptides	*Significant Peptides *p* < 0.05	Significant 10lgP^*^
Aggrecan	15	1	5	20	5	12	2	0	0
Biglycan	11	4	8	13	3	10	4	1	1
COMP	7	5	7	18	7	12	49	40	42
Decorin	5	1	3	3	1	3	3	1	1
Fibromodulin	7	1	7	18	5	12	3	1	3

Table shows unique peptides identified following either crude proteoglycan digestion using MMP‐3 or ADAMTS‐4 or IL‐1β stimulation of cartilage explants.

^#^ Significant peptides were identified by Mascot with the probability that a match was correct (*p* < 0.05) derived from the ion score.

^*^ Significant peptides from PEAKS® analysis had a 10lgP of greater than 20; this equated to a *p* value of 0.01. For IL‐1β stimulated samples, the neopeptides were only counted once if identified in multiple samples.

**Table 2:** Neopeptides Identified Following A. ADAMTS‐4 Digestion of Crude Proteoglycan Was Identified With LC‐MS/MS Using Mascot

A						
Protein	Amino Acid Before Peptide Residue	Peptide Sequence	Amino Acid Residue After	Significant MASCOT Identified Peptides; *p *< 0.05	Significant PEAKS 10lgP	Position
Aggrecan	R	LATTGQL**Y** ^#^	**L**	*	*	280–287
	R	VSLPNYPAIPTDATLELQ**N** ^#^	**L**		*	105–122
	R	LATTGQLYLA**W** ^#^	**Q**		*	280–290
	**V**	**E**DISGFPSGG**E** ^#^	**V**		*	989–998
	R	WSDGHSLQFE**N** ^#^	**W**		*	2275–2285
	**P**	**W**ATEVPSASEKPSPS**E**	**E**			787–802
	R	VSLPNYPAIPTDATL**E**	**L**			104–119
	R	**Y**DAICYTGEDFVDIPENFFA	**V**			344–363
	**D**	**L**SGTSGRA**D**	**V**			1762–1771
	**Q**	**E**AGEGPSGIL**E**	**L**			1838–1848
	**F**	**R**GQPSEGSVSG**L**	**P**			834–845
	**G**	**E**GPSGILELSGAHS**G**	**A**			1841–1855
	**I**	**H**DLVSSAMSGSG**E**	**P**			1698–1710
	**G**	**F**SGTTSGIHDLVSSAMS**G**	**S**			1702–1716
	**A**	**S**GVEDLGGLPSGGEIHLEPTASG**V**	**E**			967–988
Biglycan	K	NHLVEIPPNLP**S** ^#^*^1^	**S**	*	*	152–163
	K	LLQVVYL**H** ^#^	**T**	*	*	305–312
	**N**	**G**ISLFNNPVP**Y** ^#^	**W**	*	*	338–348
	**N**	CIEMGGNPLENSGFQPGAFDGLK*^2^*^3^	**L**	*		191–213
	K	DLPETLNELHLDH**N**	**K**		*	230–243
	**F**	**N**NPVPYWEVQPAT**F**	**R**		*	343–356
	**A**	**I**ELEDLL**R**	Y		*	248–255
	**F**	**N**NPVPYWEVQP**A**	**T**		*	343–354
	**M**	**C**PFGCH**C**	**H**			67–73
	**D**	**S**LTPTFSAMCP**F**	**G**		*	58–69
	K	DLPETLN**E**	**L**			230–237
COMP	R	AFQTVVLDPEGDAQIDP**N** ^#^	**W**	*	*	531–548
	K	QVCTDIDECETGQHNCVP**N** ^#^*^3,7^	**S**	*	*	174–192
	**N**	**W**VVLNQGMEIVQT**M** ^#^	**N**	*	*	549–562
	**Q**	**C**APGSCFPGVA**C** ^#^*^4,7^	**S**	*	*	89–100
	R	WFLQHRPQVG**Y** ^#^	**I**	*	*	682–692
	K	NTVMECDACGMQP*^5^	**A**		*	63–75
	R	CEACPPGYSGPTHEGVGMAF*^6,7^	**A**		*	149–168
Decorin	R	IHEVLDLEPLGPVCP**F** ^#^	**R**	*	*	42–57
	K	NLHALILV**N**	**N**			107–117
	K	ASYSGVSLFSNPVQYWEIQP**S**	**T**		*	323–343
	K	YIQVVYL**H**	**N**		*	294–301

B						
Protein	Amino Acid Before Peptide Residue	Peptide Sequence	Amino Acid Residue After	Significant MASCOT Identified Peptides; *p* < 0.05	Significant PEAKS 10lgP	Position
Decorin	**Q**	**M**IVVELGTNPLKSSG*^3^	**I**			177–191
Fibromodulin	R	DCPQECDCPPNFPTAM**Y** ^#^	**C**	*	*	75–91
	R	LSHNSLTN**N** ^#^	**G**		*	275–283
	R	IPPVNTNLE**N** ^#^	**L**		*	308–317
	R	INEFSISSF**C**	**T**		*	325–334
	R	KVPDGLPSALEQLYLEHNNV**Y**	**S**		*	237–257
	R	ELHLDHN*^1^	**Q**		*	180–186
	R	KVPDGLPSALEQLYLEHNNVYSVPDS**Y**	**F**		*	237–263

In the peptide sequence column, * denotes neopeptides produced at known cleavages sites to specific proteases at the N or C terminal end and **^#^** denotes neopeptide that have been identified following protease digestion with both ADAMTS‐4 and MMP‐3. A significant PEAKS® derived 10lgP value is >20 that equates to *p* value <0.05. Potential cleavage sites are marked in bold. Position refers to the amino acid of the peptide sequence within the protein.

*^1^ Ref.^8^;*^2^ Ref^15^; *^3^ 45; *^4^Ref.^7^; *^5^Ref.^6^; *^6^Ref.^46^; *^7^Ref.^49^.

**Table 3:** Neopeptides Identified Following MMP‐3 Digestion of Crude Proteoglycan Were Identified With LC‐MS/MS Using Mascot

A						
Protein	Amino Acid Before	Peptide Sequence	Amino Acid After	Significant MASCOT Identified Peptides; *p* < 0.05	Significant PEAKS 10lgP	Position
Aggrecan	R	VSLPNYPAIPTDATLELQ**N** ^#^	**L**	*	*	105–122
	R	LATTGQL**Y** ^#^	**L**		*	280–287
	R	VSLPNYPAIPTDATL**E** ^#^	**L**		*	104–119
	R	LATTGQLYLA**W** ^#^	**Q**		*	280–290
	**V**	**E**DISGFPSGG**E** ^#^	**V**			989–998
	R	TYGVRPSSETYDVY**C**	**Y**	*	*	557–571
	R	ACLQNSAIIATPEQ**L**	**Q**	*	*	174–188
	R	ITCTDPAS**Y**	**K**	*	*	2419–2427
	R	ITCTDPA**S**	**Y**	*		2419–2426
	K	GTVACGDPPVVE**H**	**A**		*	2328–2340
	**Y**	**L**AWQSGMDM**C**	**S**		*	288–297
	R	WSDGHSLQFENW**R**	**P**			2275–2287
	R	YDAICYTGEDFVDIPEN*^8^	**F**		*	344–380
	**Y**	**Q**LPFTC**K**	**K**			2320–2326
	**N**	**S**AIIATPEQ**L**	**Q**			179–188
	R	PSSETYDVY**C**	**Y**			562–571
	R	TYGIRD**T**	**N**			232–238
	K	GEWNDVPCN**Y**	**Q**		*	2310–2319
	R	VSLPNYPAIPT**D**	**A**		*	104–115
	**M**	**E**GLETSASGAEDLSG**L**	**P**			1459–1474
Biglycan	K	LLQVVYL**H** ^#^	**S**	*	*	305–312
	K	NHLVEIPPNLPS**S** ^#^*^1^	**L**		*	152–164
	**N**	**G**ISLFNNPVP**Y** ^#^*^3^	**W**			338–348
	K	VGVNDFCPVGFG**V**	**K**	*	*	319–331
	**Y**	**N**GISLFNNPVP*^9^	**Y**	*	*	337–347
	K	VGVNDFCPV**G**	**F**		*	319–28
	K	DLPETLN**E**	**L**		*	230–237
	R	NMNCIEMGGNPL**E**	**N**		*	188–200
	**P**	GVFSGLR**N**	**M**		*	181–188
	K	NHLVEIPPNLP**S** ^#^	**S**		*	152–163
	K	LLQVVY**L**	**H**			305–311
	**N**	**G**ISLFNNPVPY**W**	**E**		*	338–349
	**P**	**Y**WEVQP**A**	**T**			348–354
COMP	**N**	**W**VVLNQGMEIVQT**M** ^#^	**N**	*	*	549–562
	K	QVCTDIDECETGQHNCVP**N** ^#^*^3,7^	**S**	*	*	174–192

B						
Protein	Amino Acid Before	Peptide Sequence	Amino Acid After	Significant MASCOT Identified Peptides; *p* < 0.05	Significant PEAKS 10lgP	Position
COMP	R	AFQTVVLDPEGDAQIDP**N** ^#^	**W**	*	*	531–548
	**Q**	**C**APGSCFPGVA**C** ^#^*^4,7^	**T**		*	89–100
	R	WFLQHRPQVG**Y** ^#^	**I**		*	682–692
	R	NALWHTGDTAS**Q**	**V**	*	*	650–661
	K	QVCTDIDECETGQH**N**	**C**	*	*	174–188
	K	QMEQTYWQAN**P**	**F**	*	*	614–624
	R	AFQTVVLDPEGDAQIDPNWVVLNQGM**E**	**I**	*	*	531–557
	R	NALWHTGDTA**S**	**Q**		*	650–659
	R	AFQTVVLDPEGDAQIDPN**W**	**V**		*	531–549
	R	AFQTVVLDPEGDAQIDPNWVVL**N**	**Q**		*	531–553
	**N**	**W**VVLNQGM**E**	**I**			549–557
	R	AFQTVVLDPEGDAQIDPNWVVLNQGMEIVQTM**N**	**S**			531–563
	**N**	**S**VCINT**Q**	**C**			193–199
	**C**	**F**SQENIIWA**N**	**L**			725–734
	**I**	**D**PNWVVLNQGMEIVQTMNSDPGLAVGYT**A** ^c^	**F**			546–574
	**N**	**W**VVLNQGMEIVQTM**N**	**S**			549–563
Decorin	R	IHEVLDLEPLGPVCP**F** ^#^	**R**	*	*	42–57
	K	AVFNGLN**Q**	**M**		*	169–176
	K	ISPGAFTPL**V**	**K**		*	121–130
Fibromodulin	R	DCPQECDCPPNFPTAM**Y** ^#^	**C**	*	*	75–91
	K	IPPVNTNLE**N** ^#^	**L**		*	308–317
	R	LSHNSLTN**N** ^#^	**G**		*	275–283
	R	INEFSISSFCTVVDVM**N**	**F**	*	*	325–341
	R	LSHNSLTNNGLA**S**	**N**	*	*	275–287
	R	KVPDGLPSALEQ**L**	**Y**	*	*	237–249
	R	SLILLDLSYN**H**	**L**	*	*	224–234
	R	KVPDGLPSAL**E**	**Q**		*	237–247
	R	SAMPADAPL**C**	**L**		*	358–367
	R	INEFSISSF**C**	**T**		*	325–334
	K	YVYFQNNQI**S**	**S**			109–118
	**A**	**L**EQLYLEHNNV**Y**	**S**			246–257
	K	YVYFQNNQIS**S**	**I**			109–119
	R	KVPDGLPS**A**	**L**		*	237–245
	R	LSHNSLTNN**G**	**L**		*	257–284
	R	KVPDGLP**S**	**A**			237–244
	R	KVPDGLPSALEQLYLEHNNVYSVPD**S**	**Y**			237–262
	R	SAMPADAPLC**L**	**R**			358–368

In the peptide sequence column, * denotes neopeptides produced at known cleavages sites to specific proteases at the N or C terminal end and **^#^** denotes neopeptide that have been identified following protease digestion with both ADAMTS‐4 and MMP‐3. A significant PEAKS® derived 10lg *p* value is >20 that equates to *p* value <0.05. Potential cleavage sites are marked in bold. Position refers to the amino acid of the peptide sequence within the protein.

*^1^Ref.^8^; *^2^Ref.^15^; *^3^Ref.^45^; *^4^Ref.^7^; *^5^Ref.^6^; *^6^(Holden P, personal communication); *^7^Ref.^49^; *^8^Ref.^42^; *^9^Ref.^44^.

### Identification of Neopeptides Following IL‐1β Stimulation of Articular Cartilage Explants

Each donor was analysed separately. In control media, we identified 141 ± 4.7 and in the IL‐1β‐treated media 226 ± 17.6 proteins. Supplementary Table S2 contains complete lists of all peptides identified. A catalog of IL‐1β‐generated neopeptides (defined here as those identified in at least two treated donors but no controls) from each protein of interest was identified (Table 4A and B). Table 5 shows the neopeptides identified in multiple conditions in this study or those previously identified.

**Table 4:** Neopeptides Identified Following Treatment of Cartilage Explants With IL‐1β

A								
Protein	Amino Acid Before	Peptide Sequence	Amino Acid After	ADAMTS‐4 Digest	MMP‐3 Digest	Significant MASCOT	Significant PEAKS	Position
Aggrecan	**Q**	**E**AGEGPSGILE	**L**	✓				1838–1848
	**V**	**P**IETELPSPGEPSG	**V**					793–806
Biglycan	R	LAIQFGNY*^5^	**K**			*	*	363–370
	**P**	**Y**WEVQPATFRCVTDRLAIQ*^9^	**F**					348–366
COMP	R	DVDHDFVGDACDSDQDKDG**D**	**G**			*	*	418–437
	R	DVDHDFVGDACDSDQ**D**	**K**			*	*	418–433
	R	CEACPPGYSGPTHEGVGM*^3^	**A**			*	*	149–166
	R	CEACPPGYSGPTHEGVGMAF*^6^	**A**	✓		*	*	149–168
	R	FCPDGTPSPCHEKA**D**	**C**			*	*	226–240
	K	NTVMECDACGMQP*^3^	**A**	✓		*	*	63–75
	R	AFQTVVLDPEGDAQIDPNWVVLNQGMEIVQTM**N**	**S**		✓			531–563
	K	QMEQTYWQ**A**	**N**			*	*	614– 622
	R	LVPNPGQEDADRDGVG**D**	**V**				*	484–500
	R	LGVFCFSQENIIWA**N**	**L**		✓			720–734
	**A**	**Q**CAPGSCFPGVA**C**	**T**	✓	✓		*	88–100
	R	NALWHTG**D**	**T**				*	650–657
	**F**	**C**FSQENIIW*^4^	**A**					724–732
	R	SCVCAVGWAGNG**L**	**L**					250–262
	R	NAVDNCPRVPNSDQKDSD**G**	**D**					380–398
	K	QVCTDIDECETGQH**N**	**C**		✓	*	*	174–188
	R	SCVCAVGWAGNGLL**C**	**G**			*	*	250–264
	**L**	**A**QCAPGSCFPGVACTQ*^5,3^	**T**	✓	✓	*	*	87–102
	R	KDNCVTVPNSGQEDADRDGIGD*^3^	**A**			*	*	287–308
	R	VSVRPLAQCAPGSCFPGV**A**	**C**			*	*	81–99
	R	VSVRPLAQCAPG**S**	**C**			*	*	81–93
	**Q**	**C**APGSCFPGVACTQTA*^4^	**S**			*	*	89–104
	R	LGVFCFSQE**N**	**I**			*	*	720–729
	R	VSVRPLAQ*^4,5^	**C**			*	*	81–88
	R	VSVRPLAQ**C**	**A**			*	*	81–89

B								
Protein	Amino Acid Before	Peptide Sequence	Amino Acid After	ADAMTS‐4 Digest	MMP‐3 Digest	Significant MASCOT	Significant PEAKS	Position
	R	AFQTVVLDPEGDAQIDPNWVVLN**Q**	**G**			*	*	531–554
	**Q**	**C**APGSCFPGVAC*^4,7^	**T**			*	*	89–100
	**Q**	**C**APGSCFPGVACTQ*^4^	**T**			*	*	89–102
	K	QVCTDIDECETGQHNCVP**N**	**S**		✓	*	*	174–192
	R	VPNSDQKDSDG**D**	**G**			*	*	388–399
	**Q**	**C**APGSCFPGVACT*^4^	**Q**			*	*	89–101
	R	VSVRPLAQCAPGSCFPGVACTQ**T**	**A**			*	*	81–103
COMP	R	LGVFCFSQ**E**	**N**			*	*	720–728
	R	KDNCVTVPNSGQE**D**	**A**			*	*	287–300
	R	SCVCAVG**W**	**A**			*	*	250–257
	R	SCVCAVGWAGNGL**L**	**C**			*	*	250–263
	R	NALWHTGDTAS**Q**	**V**		✓	*	*	650–661
	R	SCVCAVGWAG**N**	**G**			*		250–260
	**C**	**A**VGWAGNGLL**C**	**G**			*	*	254–264
	R	NAVDNCPRVPNS**D**	**Q**			*	*	380–392
	R	FYEGPELVADSNVVL**D**	**T**			*	*	697–712
	**Q**	**C**APGSCFPGVA*^4^	**C**			*	*	89–99
	R	LVPNPGQEDADR**D**	**G**			*	*	484–496
	**M**	**E**CDACGMQPAR	T			*	*	67–77
	R	DVDHDFVGDAC**D**	**S**			*	*	418–429
	R	NTDGDKWG**D**	**A**			*	*	336–344
	R	VSVRPLAQCAP**G**	**S**			*	*	81–92
	R	VSVRPLAQCAPGSCFPGVA**C**	**T**				*	81–100
	**A**	**V**GWAGNGLLCGR	D			*	*	255–266
Decorin	K	KASYSGVSL**F**	**S**				*	322–331
	**Q**	**M**IVVELGTNPLKSSG*^3^	**I**	✓				177–191
	**P**	**P**GLPPSLTE**L**	**H**			*		218–227
Fibromodulin	R	KVPDGLPSALEQ**L**	**Y**		✓		*	237–249
	R	INEFSISSFCTVVDV**M**	**N**			*	*	325–340
	R	KVPDGLPSALEQLYLEHNNV**Y**	**S**	✓	✓		*	237–257

Table indicates neopeptides identified in at least two donor samples. Neopeptides also evident in the ADAMTS‐4 and MMP‐3 experiments are also indicated with a tick. * indicates neopeptides produced at known cleavages sites to specific proteases. Peptides with a significant (*p* < 0.05) match that the sequence was correct based on Mascot ion scores are indicated with a black asterisk. A significant PEAKS® derived 10lg *p* value is >20; equating to a *p*‐value <0.05. Position refers to the amino acid of the peptide sequence within the protein.

*^4^Ref.^7^; *^5^Ref.^6^; *^6^(Holden P, personal communication); *^7^Ref.^49^ *^9^Ref.^44^

**Table 5:** Summarised Neopeptides Data

Protein	Amino Acid Before Peptide Residue	Peptide Sequence	Amino Acid Residue After	ADAMTS‐4 Digest	MMP‐3 Digest	IL‐1 Treatment	Position
Aggrecan	**V**	**E**DISGFPSGG**E**	**V**	✓	✓		989–998
	R	LATTGQLYLA**W**	**Q**	✓	✓		280–290
	R	LATTGQL**Y**	**L**	✓	✓		280–287
	R	VSLPNYPAIPTDATLELQ**N**	**L**	✓	✓		105–122
	R	VSLPNYPAIPTDATL**E**	**L**	✓	✓		105–119
	R	WSDGHSLQFE**N**	**W**	✓	✓		2275–2285
	R	YDAICYTGEDFVDIPEN*^8^	**F**		✓		344–380
	**Q**	**E**AGEGPSGIL**E**	**L**	✓		✓	1838–1848
Biglycan	**N**	**C**IEMGGNPLENSGFQPGAFDGLK*^2^*^3^	L	✓			191–213
	**N**	**G**ISLFNNPVPY*^3^	**W**	✓	✓		338–348
	K	LLQVVYL**H**	**T**	✓	✓		305–312
	K	NHLVEIPPNLPSS*^1^	**L**	✓	✓		152–164
	K	NHLVEIPPNLP**S**	**S**	✓	✓		152–163
	K	VGVNDFCPVGFG**V**	**K**		✓		319–328
	**Y**	**N**GISLFNNPVP*^9^	**Y**		✓		337–347
	R	LAIQFGNY*^5^	**K**			✓	363–370
	**P**	**Y**WEVQPATFRCVTDRLAIQ*^9^	**F**			✓	348–366
COMP	**L**	AQCAPGSCFPGVACTQ*^5,3^	**T**	✓	✓	✓	87–102
	R	AFQTVVLDPEGDAQIDPNWVVLNQGMEIVQTM**N**	**S**		✓	✓	531–563
	R	AFQTVVLDPEGDAQIDP**N**	**W**	✓	✓		531–548
	**Q**	**C**APGSCFPGVA*^4^	**C**			✓	89–99
	**Q**	**C**APGSCFPGVAC*^4,7^	**T**	✓	✓	✓	89–100
	**Q**	**C**APGSCFPGVACT*^4^	**Q**			✓	89–101
	**Q**	**C**APGSCFPGVACTQ*^4^	**T**			✓	89–102
	**Q**	**C**APGSCFPGVACTQTA*^4^	**S**			✓	89–104
	R	CEACPPGYSGPTHEGVGMAF*^6^	**A**	✓		✓	149–168
	**F**	**C**FSQENIIW*^4^	**A**			✓	724–732
	R	KDNCVTVPNSGQEDADRDGIGD*^3^	**A**			✓	287–308
	R	LGVFCFSQENIIWA**N**	**L**		✓	✓	720–734
	R	NALWHTGDTAS**Q**	**V**		✓	✓	650–661
	K	NTVMECDACGMQP*^5^	**A**	✓		✓	63–75
	K	QVCTDIDECETGQHNCVPN*^3,7^	**S**	✓	✓		174–192
	K	QVCTDIDECETGQH**N**	**C**		✓	✓	174–188
	R	VSVRPLAQ*^4,5^	**C**			✓	81–88
	R	WFLQHRPQVG**Y**	**I**	✓	✓		682–692
Decorin	R	IHEVLDLEPLGPVCP**F**	**R**	✓	✓		42–57
	**Q**	**M**IVVELGTNPLKSSG*^3^	**I**	✓		✓	177–191
Fibromodulin	R	DCPQECDCPPNFPTAM**Y**	**C**	✓	✓		75–91
	R	ELHLDHN*^1^	**Q**	✓			180–186
	K	IPPVNTNLE**N**	**L**	✓	✓		308–317
	R	KVPDGLPSALEQLYLEHNNV**Y**	**S**	✓	✓	✓	237–257
	R	KVPDGLPSALEQ**L**	**Y**		✓	✓	237–249
	R	LSHNSLTN**N**	**G**	✓	✓		275–283

Position refers to the amino acid of the peptide sequence within the protein.

*Neopeptides produced at known cleavages sites to specific proteases.

## DISCUSSION

We have performed a proteomic analysis of crude proteoglycan extract identifying protease‐related neopeptides within the cartilage proteoglycan‐enriched fraction and compared these to neopeptides produced in an IL‐1β driven in‐vitro early inflammatory model of OA. OA is a syndrome, and it is likely that not all forms of OA are directly related to IL‐1. Recent work has disputed a substantial role for IL‐1 in OA. There is no change in the concentration of IL‐1β in OA synovial fluid,^26^ the use of IL‐1β receptor antagonist has had variable results,^27^, ^28^ the role in murine OA is also disputed.^29^, ^30^ In addition, most in‐vitro experiments use super physiological concentrations of IL‐1β (synovial fluid concentrations are 10 pg/ml). Thus, while a role can be argued for IL‐1β in OA achieving, clinical relevance may require additional factors in in‐vitro studies. However, we feel that IL‐1 is still relevant cytokine to study in relation to many pathogenesis's of OA.^31^ Some of the neopeptides were generated by both proteases and in the Il‐1β‐treated media, whereas others were unique for either a single protease or Il‐1β dependant. Furthermore, a number of previously identified neopeptides or cleavage sites pertaining to these were evident.

We hypothesise that a number of the neopeptides identified contain cleavage sites due to protease degradation, either MMP‐3 or ADAMTS‐4 or proteases downstream of Il‐1β. These proteases have been implicated in the pathogenesis of OA.^3^, ^14^, ^31^, ^32^, ^33^ We were interested in important cartilage proteins with diverse functions, including key regulators of collagen fiber assembly; the small leucine‐rich proteoglycans; bigycan, decorin, fibromodulin, and COMP. In addition, we studied aggrecan neopeptides as this is the major proteoglycan in cartilage, enabling the tissue to resist compressive loads. Therefore, rather than extracting proteins from whole cartilage, a crude proteoglycan extract was prepared. Classic methodologies, previously used to identify cartilage fragments in aggrecan,^34^ were used in order to remove proteins of non‐interest, thus reducing sample complexity for downstream LC‐MS/MS. The A1–A2 fractions contained the proteoglycans of interest, including aggrecan as determined by immunoblotting. Subsequent analysis at the protein level demonstrated that the abundant proteins in the crude proteoglycan extract were the ECM proteins in which we were interested.

We used equine samples in this study as the horse is an athletic animal and is considered an excellent animal model for human joint diseases due to extensive knowledge of its pathogenesis and clinical experience of the disease.^35^ Equine tissue was readily obtained, enabling collection of cartilage samples from macroscopically normal horses.

Equine recombinant proteins relevant to this study were not freely available, so human recombinant proteins were used. The BLAST tool predicted 89% sequence homology between equine and human for the catalytic domain of MMP‐3 and 99.6% homology for ADAMTS‐4 truncated recombinant protein. Subsequently, immunoblotting was used to validate our approach. When the crude proteoglycan was digested with ADAMTS‐4 ARGXX, cleavage sites were evident but no DIPEN cleavages. In contrast, when digestion was undertaken with MMP‐3 DIPEN but not ARGXX cleavage sites were seen.

ADAMTS‐4 and MMP‐3 had various preferences for ECM proteins; ADAMTS‐4 generated the most numerous neopeptides from proteolysis of aggrecan and biglycan, whereas except for decorin, MMP‐3 produced similar numbers of neopeptides for all proteoglycans and COMP. Significantly more neopeptides were identified in aggrecan than in previous studies.^5^ Aggrecan is the first matrix component to undergo measurable loss in the progression of OA,^32^ which has been previously principally attributed to ADAMTS‐4 and ADAMTS‐5 cleavage.^36^ The profile of neopeptides produced was greatest in the media of IL‐1β stimulated explants. This may be due to the less complex digest obtained from a media compared to a crude proteoglycan extract, thus reducing sample complexity and increasing the number of neopeptides identified, especially medium to low abundance ones. In addition, it is probably due to the non‐specific nature of the numerous MMPs (and other proteases) upregulated following Il‐1β stimulation.^37^ Of the two protease digestions, MMP‐3 produced the greatest number of neopeptides from crude proteoglycan extract in agreement with other studies.^5^

There were examples of both ADAMTS4 and MMP3 generating identical neopeptides, for example the biglycan peptide ^337^GISLFNNPVPY and the COMP neopeptide ^89^CAPGSCFPGVAC. This could indicate that under some conditions, the two proteinases have the same specificity. Although an alternative hypothesis would be that this is due to the activation of ADAMTS4 by MMP3‐mediated C‐terminal processing of ADAMTS4, such as previously suggested for MMP17.^38^

Of the proteins investigated, COMP had the most neopeptides identified following Il‐1β stimulation of cartilage explants. This could be due to a number of factors. COMP, a pentomeric protein has a relatively even distribution of arginines and lysines within its sequence, making it an ideal for tryptic digestion producing tryptic (or semi‐ or none‐tryptic in the case of neopeptides) masses of optimum mass range for detection. While from our previous IL‐1β explant studies, in molar concentration terms, it is the most abundant protein.^21^ It also has few post‐translational modifications, such as glycosylations. These peptides would not be identified with the protein identification software used in this study. Finally COMP has been investigated as a musculoskeletal biomarker of OA^39^ and tendon injury^7^ in numerous studies and it is hypothesized that in arthritis, it is subject to extensive degradation accounting for the large number of neopeptides identified.

Our workflow used a mass spectrometer with high resolution and high mass/charge (m/z) accuracy. For data processing, we used both the MASCOT search engine that uses of probabilistic search algorithms and the software PEAKS® enabling significantly improved sensitivity and through algorithms that enable sequencing de‐novo.^40^ As the aim of our approach was to provide a “first pass” list of peptides for further investigation, it was important that peptide identification results were statistically validated to avoid false positives. Therefore, in the PEAKS® analysis, we employed the decoy‐fusion method that joins the decoy and target sequences of the same protein together as a “fused” sequence. The decoy fusion method avoids some pitfalls in the standard target‐decoy method, and is more conservative.^40^

We used the enzyme trypsin to produce high cleavage specificity peptides of suitable charge and sequence length for MS. Trypsin cleaves by the recognition of a target amino acid in a binding pocket. A negatively charged aspartate residue at the bottom of this pocket limits the amino acids to which this enzyme will recognize to arginine (R) and lysine (K) due to their long basic side chains with a few exceptions.^41^ Thus, without further protease digestion, C‐terminal R or K peptides are produced. Other possible fragmentations are produced by other proteases either from the environment (exogenous proteases that remove amino acids at the peptide terminals), the sample itself (for example following protease treatment) or following auto proteolysis (by intracellular enzymes and/or enzymes not normally active in the tissue) during the period between death and the addition of proteinase inhibitors. In order to minimize the latter, samples were collected by the removal of the leg distal to the carpus at the time of death and the cartilage removed within 2 h of death. Hence, semi‐tryptic peptides or none tryptic peptides will possess a cleavage pattern independent of trypsin. These peptides are then subject to further fragmentation in the MS in order to identify the sequence of amino acids through the use of search engine specific algorithms. Thus, a workflow based on LC‐MS/MS, de‐novo sequencing and database searching provide an accurate and convenient method to identify peptide products released by either specific proteases or following cytokine stimulation.

A number of neopeptides pertaining to previously identified cleavage sites were evident. The neopeptide ^344^YDAICYTGEDFVDIPEN corresponding to the DIPEN neoepitope^42^ was demonstrated and confirmed with immunoblotting. However, the other major aggrecanase‐derived cleavage sites (reviewed^43^) in aggrecan were not identified. This may be due to the size of the peptides produced being outside the mass range of the instrument. For instance, cleavage at PTPFKEEE^1745^ ^1746^GLGSVELSG would produce a small peptide, K.EEE that is below the mass range identifiable and GLGSVELSGLPSGDADLSGTSGR whose mass would be too great for detection. Furthermore, neopeptides corresponding to other previously identified cleavage sites were evident in our data; six for MMP‐3 treatment,^7^, ^8^, ^42^, ^44^, ^45^ eight following ADAMTS‐4 treatment^6^, ^7^, ^8^, ^15^, ^45^ (Holden P, personal communication), and 15 in equine Il‐1β model^6^, ^7^, ^44^, ^45^ (Holden P, personal communication).

We studied the sequence context of the neopeptides within the corresponding protein. Interestingly, apart from decorin, where neopeptides identified were equally distributed throughout the protein, neopeptides generated from COMP, biglycan, and fibromodulin were primarily from the C‐terminal region. This has previously been described for COMP.^5^ This dominance of neopeptides with cleavage identified at the C terminus could be due to the structure and dynamics of the substrate protein responsible for the targeting in the enzyme molecular recognition system.^46^ Thus, there are likely sites of limited proteolysis that may account for our findings.

One of the limitations of this study is the lack of validation of candidate neopeptides. This would require the development of monoclonal epitope antibodies that was beyond the scope of this study. We were only able to validate the aggrecan neopeptide DIPEN. In addition, for some neopeptides, PEAKS® analysis was significant; whereas in Mascot, it was not (and visa‐versa). We identified neopeptides with significant scores. This gives a probability that the sequence established by Mascot or PEAKS® was correct and the peptide mass and subsequent fragmentation pattern/de‐novo sequencing were not due to another peptide. However, this is only a probability and some neopeptides may be either missed or falsely identified. An example of this is the identification of the aggrecan neopeptide ^1838^EAGEGPSGILE. The expected aggrecanase‐mediated cleavage is at ^1839^AGEGPSGILE. This neopeptide has one potential chondroitin sulphate attachment site at SG and the workflow used would not fully remove this glycosaminoglycan side chain. The presence of a CS would significantly alter the mass of the neopeptide and thus preclude identification. Although it is likely that there is a CS at this site, we cannot be totally confident about this without undertaking further work. If a CS was present here, it would change the mass of the peptide and not be identified by our analysis.

Although protease inhibitors were present throughout our workflow, it is possible that some of the peptides identified were cleaved by endogenous proteases. This is especially likely where peptides are seen with successive amino acids removed, such as the exoproteolytic fraying of ^152^NHLVEIPPNLPSS to ^152^NHLVEIPPNLPS produced following MMP3 treatment in biglycan. Most peptides produced by endogenous proteases were eliminated from the data analysis as peptides identified in both control and protease‐treated conditions were excluded.

Other potential anomalies in the data are regarding miscleaves due to the digestion enzyme trypsin. Trypsin cleaves the carboxy‐terminal peptide bond of both Arg and Lys. However, cleavage by trypsin is not always reproducible or predictable. For example, one of the most usual mistakes is the omission of a cleavable residue (miscleavage). For trypsin, such miscleaved positions can be predicted when an Arg/Lys is followed by Pro, when successive Lys/Arg or positive charges are close to each other, and if several Asp/Glu are close to the positively charged residue. For example, the decorin neopeptide following MMP3 and ADAMTS4 treatment ^42^IHEVLDLEPLGPVCPF.R. This semitryptic neopeptide (at the C terminus) is cleaved between Phe and Arg. This could be due to a miscleave due to the presence of upstream Pro.

There is a potential for some unique neopeptides identified to act as markers of specific proteases, cartilage degradation, and to measure ECM breakdown in other tissues.^6^, ^7^, ^44^ Until recently, the progression of OA has been measured almost exclusively by radiography to assess space narrowing, or by magnetic resonance imaging of the joint. There is a need to progress to translatable biomarkers with the ability to monitor protease activity and OA disease. The peptide fragments demonstrated in this study could serve as early indicators of cartilage turnover similar to others studied in COMP^47^ and aggrecan^48^ by providing candidates for assays in animal studies and clinical settings, not only in cartilage but wherever these proteins are expressed, such as tendon, ligament, muscle, and liver. In our recent synovial fluid study, we identified some of the neopeptides identified in this study in synovial fluid from OA but not normal horses,^45^ providing further evidence that some of the neopeptides identified here could provide biomarkers. Indeed the neopeptides identified in this study and in early OA synovial fluid would be ideal candidates for future studies to delineate degradative mechanism in‐vivo. For example, the biglycan neopeptides ^348^YWEVQPATFRCVTDRLAIQ and ^191^CIEMGGNPLENSGFQPGAFDGLK and the COMP neopeptide ^174^QVCTDIDECETGQHN are well supported in the literature and may be the candidates for raising monoclonal neo‐epitope antibodies. Furthermore, a number of biglycan, aggrecan, COMP, decorin, and fibromodulin neopeptides following Il‐1β treatment of cartilage explants, and/or ADAMTS4 or MMP3 treatment of cartilage crude proteoglycan extract were identified for the first time. Many of the sequences are highly conserved between equine, human, and bovine. These are potential candidates for generating antibodies that could assist in elucidating the role of proteases in not only cartilage matrix protein turnover but also in other tissues, such as tendon. Furthermore, with ever increasing elegant mass spectrometry techniques becoming accessible in the future, it may be possible to use defined sets of the neopeptides themselves as markers of cartilage degradation and OA. Our neopeptides could lead to novel methods for OA biomarker pattern identification, validation, and more importantly OA patient stratification data to facilitate appropriate OA diagnosis and treatment.

## CONCLUSIONS

Improved knowledge of specific peptide fragments and the proteases generating these fragments will aid in the identification of markers of ECM degradation such as in OA.

## AUTHORS' CONTRIBUTIONS

M.J.P. conceived the study, carried out all the laboratory work and analysis, and drafted the manuscript. P.D.C. and D.J.T. participated in the design and coordination of the study and helped to draft the manuscript. All authors read and approved the final manuscript.

## Supporting information

Additional supporting information may be found in the online version of this article.

Supplementary Table S1.

Supplementary Table S2.
